# Green Synthesis of Magnesium Oxide Nanoparticles by Using *Abrus precatorius* Bark Extract and Their Photocatalytic, Antioxidant, Antibacterial, and Cytotoxicity Activities

**DOI:** 10.3390/bioengineering10030302

**Published:** 2023-02-27

**Authors:** Saheb Ali, Kattakgoundar Govindaraj Sudha, Natesan Thirumalaivasan, Maqusood Ahamed, Saravanan Pandiaraj, Vijayarangan Devi Rajeswari, Yamini Vinayagam, Muthu Thiruvengadam, Rajakumar Govindasamy

**Affiliations:** 1 Department of Periodontics, Saveetha Dental College and Hospitals, Saveetha Institute of Medical and Technical Sciences (SIMATS), Saveetha University, Chennai 600077, Tamil Nadu, India; 2 Department of Biotechnology, K. S. Rangasamy College of Arts and Science (Autonomous), Tiruchengode 637215, Tamil Nadu, India; 3 Department of Physics and Astronomy, College of Science, King Saud University, Riyadh 11451, Saudi Arabia; 4 Department of Self-Development Skills, CFY Deanship, King Saud University, Riyadh 11451, Saudi Arabia; 5 Department of Biomedical Sciences, School of Bioscience and Technology, Vellore Institute of Technology, Vellore 632014, Tamil Nadu, India; 6 Department of Applied Bioscience, College of Life and Environmental Sciences, Konkuk University, Seoul 05029, Republic of Korea; 7 Department of Orthodontics, Saveetha Dental College and Hospitals, Saveetha Institute of Medical and Technical Sciences (SIMATS), Saveetha University, Chennai 600077, Tamil Nadu, India

**Keywords:** *Abrus precatorius*, MgO nanoparticles, biomedical engineering, cytotoxicity, genotoxicity, protein signaling pathways

## Abstract

The current research is concerned with the synthesis of magnesium oxide (MgO) nanoparticles (NPs) from *Abrus precatorius* L. bark extract via the green chemistry method. The synthesized MgO NPs was confirmed by using several characterization methods like XRD, FTIR, SEM, TEM, and UV-visible analysis. The synthesized MgO NPs displayed a small particle size along with a specific surface area. *Abrus precatorius* bark synthesized MgO NPs with a higher ratio of dye degradation, and antioxidant activity showed a higher percentage of free radical scavenging in synthesized MgO NPs. Zebrafish embryos were used as a model organism to assess the toxicity of the obtained MgO nanoparticles, and the results concluded that the MgO NPs were nontoxic. In addition, the anticancer properties of MgO nanoparticles were analyzed by using a human melanoma cancer cell line (A375) via MTT, XTT, NRU, and LDH assessment. MgO NPs treated a human melanoma cancer cell line and resulted in apoptosis and necrosis based on the concentration, which was confirmed through a genotoxicity assay. Moreover, the molecular mechanisms in necrosis and apoptosis were conferred to depict the association of magnesium oxide nanoparticles with the human melanoma cancer cell line. The current study on MgO NPs showed a broad-scope understanding of the use of these nanoparticles as a medicinal drug for melanoma cancer via its physiological mechanism and also a novel route to obtain MgO NPs by using the green chemistry method.

## 1. Introduction

Cancer is the most obvious form of fetal disease because the cell grows uncontrollably. Melanoma is the third-most important type of skin cancer, following basal and squamous cell cancer. This type of cancer might originate from melanocytes, cells that are esoteric in the manufacture of the pigment melanin and that are subject to the color of the hair, skin, and eyes [1]. The majority of melanoma is black or brown in color, though other colors can appear. UV rays and skin phenotype were the two most important factors in the development of melanoma. Then the UV revelation shows the most likely alterable peril, and because of this, it has gained much more interest. Melanoma cancer can affect all types of skin and can be treated by removing the patient’s genetic background [2]. Almost 10% of patients are likely affected by the family origin of melanoma. Nowadays, a sequence of genes bearing melanoma preremoval mutations has been determined, but it is believed that other conductive genes are still to be identified. Each year there is a higher ratio of casualties because of some types of cancer, and in the majority of cases, targetable therapeutic substances still need to be identified [3]. Cancer therapies like radiotherapy, surgery, and chemotherapy are currently used for medicinal purposes. This therapy has different problems and also includes some restrictions. To overcome such problems, synthesized MgO NPs are manufactured, and they have shown potential effects in cancer treatment [4].

The nanotechnology field has attracted many researchers due to its broad range of applications. This field has technology combined in synthesis, characterization, and the creation of implementations from the manufactured particles, which is a minimum of one dimension on the nanoscale. Nanoparticles are structural constituents on the scale between 1- and 100-nm particles [5]. Magnesium oxide nanoparticles are attracting more interest compared with other metal oxide nanoparticles. They are effectively used in several areas, and they have interesting structural particles in biological applications because of their increased stability-to-weight ratio, less witness, better properties, are recyclable, nontoxic, and are hygroscopic in nature. These properties of MgO NPs enhance the activity and also have several applications like an increase in melting point, high cost, biocatalytic properties, and biocompatibility [6,7]. MgO NPs have a broad scope of implementation in the medicinal field, especially bone regrowth for antibacterial and antimicrobial suppression. They are also applied in cryoinjury. Based on this, it was used in the absorption of uranium ions, catalysis, lithium-ion powers, and excretion of toxic waste [8].

Until now, there has been a variety of approaches used for the synthesis of MgO NPs, like combustion, sonochemical, spray pyrolysis, and coprecipitation approaches. All of the abovementioned methods require toxic chemicals, costly instruments, and time-consuming processes. In order to overcome these difficulties, an alternative route of green chemistry approaches is used for the obtaining of magnesium oxide NPs [9,10]. Recently, there have been various biomediated materials used for green syntheses, such as bacteria, fungi, enzymes, and plants [11]. Compared with these diverse materials, plants have rich sources of active metabolites, are ecofriendly, are available in large quantities, and can be used for the large-scale formation of nanomaterials [12,13].

*Abrus precatorius*, known as the Fabaceae family and locally as a jequirity bean or rosary pea, was an herbaceous flowering plant in the bean family [13]. It is a slender, perennial climber, and its long, pinnately leafed leaves are double-surrounded by trees. This plant’s leaves are used for various medicinal applications. This plant is marked as an Indian medical codex. This plant contains flavonoids and titerpines, which are the main phytochemical agents [14]. *Abrus precatorius* extract is traditionally applied to cure tetanus and inhibit rabies. The whole plant is used in various medicinal implementations to cure scratches, sores, and wounds. The plant’s leaves are used to treat coughs, fevers, and colds. Due to the medicinal properties of this plant, we have used it for the synthesis of MgO NPs. Hence, this plant was used for the treatment of liver disease, worm infection, and blisters [15]. Therefore, we have made an alternative route to identify the application of *A. precatorius*-mediated synthesized MgO NPs. The present research was intended to synthesize MgO NPs via *A. precatorius* bark leaf extract to analyze the prospective of applying MgO NPs as an anticancer medicine and also examine the toxicity of MgO NPs via zebrafish eggs. In addition, the antioxidant, photocatalytic, cytotoxicity, genotoxicity, intracellular ROS, and cellular mechanisms of MgO NPs are also analyzed through the human melanoma cancer cell line (A375) to confirm MgO NPs as an efficient anticancer medicine.

## 2. Materials and Methods

### 2.1. Sample Preparation

The young bark of *Abrus precatorius* L. was collected from the rainforest of the Andaman and Nicobar Islands, India, of latitude 11.9761° N and longitude 92.9876° E. The obtained bark of *A. precatorius* was allowed to be shade-dried for 10 days at 37 °C. Continuing to dry reactions, the plant bark was completely washed with DD H_2_O in order to eliminate dust materials. A total of 10 g of the dried powder was diluted with the help of 100 mL and then the diluted suspension was exposed to a heating process under a hot plate (80 °C) for 3 h. After completion of the heating process, the bark extract was filtered via Whatman No. 1. Then the suspension was stored at 4 °C for further analysis [16].

### 2.2. Synthesis of MgO NPs

Green chemistry was used to create magnesium oxide nanoparticles from *A. precatorius* bark extract. The hydroxyl and carbonyl groups acted as stabilizing and reducing substances and were used as coprecipitation agents for synthesizing MgO NPs. A total of 10 mL of 0.1 M Mg(NO_3_)_2_ was added to 40 mL of *A. precatorius* aqueous bark and stirred continuously for 45 min. Following that, 6.0 mL of 0.2 M NaOH was added drop by drop to the mixture suspension to form a visible precipitate. Furthermore, the solution was allowed to remain at 25 ± 3 °C. Then the precipitates were washed three times with the help of DD water, and the dried precipitate was calcinated at 873 K for 4 h to obtain a fine pure powder [17].

### 2.3. Characterization of MgO NPs

The obtained fine form of powder was allowed for confirmation process by using different characterization techniques for the determination of the nanoparticle’s physicochemical constituents like size, structure, shape, and purity. The X-ray diffraction method (XRD) (Difray, Leninskiy, Russia) was applied for crystal structure identification, via CuKα (λ = 1.542 Å), the intent for the formation of X-ray analyzed at 40 kV and 30 mA along with scan-step of 0.02° in between 10 and 80° (293 K). Fourier transform infrared spectroscopy (FTIR) analysis of MgO NPs was identified by using an FTIR spectrometer (Spectrum 100, Perkin Elmer, Waltham, MA, USA) [18]. The frequency scale of 400–4000 cm^−1^ was used to analyze the magnification of 4 cm^−1^ at 25 ± 3 °C, which was applied to determine the functional properties formed in the sample. Scanning electron microscope-coupled energy-dispersive X-ray spectroscopy was implemented to determine the shape of elemental components by analyzing samples on higher power X-ray (Tescan Vega 3, (TESCAN, Brono-Kohoutovice, Czech Republic) with SDD—XMAS, Tokyo, Japan). A total of 0.001 g of MgO NPs was diluted into 10 mL of ethanol then the suspension was maintained under sonication for 30 min. After the sonication, the diluted suspension was kept under a carbon tube that was enabled with an SEM holder. Then the drop of the sample was thoroughly dried and the sample was analyzed by maintaining the power of 10 kV [19]. The size and shape of the *A. precatorius* bark extract-mediated obtained MgO NPs were analyzed via transmission electron microscope JEOL (TEM, JEM-2010, JEOL, Tokyo, Japan). A total of 0.03 g of synthesized MgO NPs was diluted in distilled water after the solution was sonicated for 10 min. We added a single drop of nanoparticle solution onto a carbon-coated copper grid, and then dried at room temperature. Furthermore, it was allowed for imaging of the obtained sample. The absorption wavelength was analyzed through the exposure of synthesized MgO NPs by using a UV–Vis spectrophotometer (Cary 8454; Agilent Technologies, Singapore), which was analyzed by using the visible and infrared frequency range of 180–800 nm [20].

### 2.4. Photocatalytic Activity

The photocatalytic activity was evaluated by diluting the *Abrus precatorius* L. bark extract of synthesized MgO NPs (20, 40, 60, 80, 100, and 120 μg/mL) in methylene blue (MB) dye (Himedia, Mumbai, India). In this assay, the MgO NPs 5 g/L was dispersed on 30 mg/L methylene blue dye and the suspension solution was nourished with pH 7. Afterward, the solution was subjected to the agitation process with the help of a magnetic stirrer for homogenous formation under a cubic UV chamber followed by constant UV irradiation at 40 W. The photocatalytic activity was evaluated by 1 mL of the homogenous sample intensity wavelength determined at (λ) 665 nm, and the untreated methylene blue sample alone was used as the control sample [21]. The catalytic ratio of methylene blue was analyzed by initial and end absorption via the equation mentioned below,

In (C/C₀) = −kt,

whereas starting and end absorbance was marked as C and C₀, k depicts as the catalytic ratio of MB and t was marked as time [22].

### 2.5. Antioxidant Activity

The antioxidant activity was analyzed via 2,2-diphenyl-1-picrylhydrazyl (DPPH) assay. For this assay, various ratios (20, 40, 60, 80, 100 and 120 μg/mL) of MgO NPs mixed with H_2_O were used in a dose-mediated reaction. The obtained magnesium oxide nanoparticles sample was dispersed to various ratios, and then the suspension sample was moved into vials. A total of 3 mL of DPPH solution was mixed with MgO NPs, and the mixed suspension was noted as a test sample, whereas the DPPH solution was marked as a control sample, and then the suspension samples were kept for incubation for about 30 min at 27 °C. Then the supernatant was procured via the centrifugation process at 10,000 rpm for 3 min [23,24]. The absorbance frequency was evaluated via UV visible spectrophotometer, and the amount of free radical and maximum solidity was measured through the following equation:

$$\mathrm{Antioxidant}\;\mathrm{activity}\;\left(\%\right)=\frac{\mathrm{Absorption}\;\times \;\mathrm{Test}\;\mathrm{absorption}}{\mathrm{DPPH}\;\mathrm{percentage}\;=\;\mathrm{Control}}\times 100$$

This equation shows that CA was noted as control absorbance and TA was noted as test absorbance.

### 2.6. Evaluation of in Vitro Toxicity

*A. precatorius* bark extract of obtained magnesium oxide NPs toxicity was determined by using zebrafish embryos. Various ratios of magnesium oxide nanoparticles were exposed to measure the mortality percentage in zebrafish under a marked time period and the unexposed embryos were marked as control. Based on the OECD-203 protocol, the toxicity was evaluated by using 30 marked zebrafish eggs at various ratios (control, 20, 40, 60, 80, 100 and 120 μg/mL) of MgO NPs in Hank’s solution. Then, the embryos were moved to respective wells for the development of the eyes, tail, and head. This was done by using a microscope at 40× in 24 h time duration. In this assay, the H_2_O is maintained at a sustained temperature. The mortality and viable embryos were measured for each 24-h time duration to excrete impurity on the suspension. The mortality of hatched embryos was evaluated for 24 h [25].

### 2.7. Cytotoxicity Evaluation

#### 2.7.1. MTT Assay

The cytotoxicity of obtained MgO nanoparticles was analyzed by using a human melanoma cancer cell line (A375) depending on the intake of tetrazolium salts, i.e., 3-(4,5-dimethylthiazol-2-yl)-2,5-diphenyltetraazolium bromide with different concentrations of the synthesized MgO NPs (control, 20, 40, 60, 80, 100 and 120 μg/mL). For the assay, cells were transferred to 96-well plates, and the cells were subjected to development in the incubator (5% CO_2_) for 48 h. Then 100 mL of DMSO solution were transferred to each well, and then the well was kept under incubation for 48 h. Additionally, 100 μL of MTT solution was transferred to each well and then kept for 2 h incubation, and then the viable and mortality cells were determined. The mitochondria of the cells included succinate dehydrogenase and mitochondrial enzyme (NADPH) that converts the yellow MTT salt to purple insoluble formazan because of the tetrazolium ring damage [26]. The ratio of purple MTT was determined via spectrophotometer at 570 nm, and the percentage of mortality cells was evaluated through the following equation:

$$\mathrm{MTT}\;\%\;=\frac{\mathrm{Absorption}\;\mathrm{of}\;\mathrm{control}\;\mathrm{wells}\;–\;\mathrm{Absorption}\;\mathrm{of}\;\mathrm{test}\;\mathrm{wells}}{\mathrm{Absorption}\;\mathrm{of}\;\mathrm{control}\;\mathrm{wells}}\times 100$$

#### 2.7.2. XTT Assay

A (2,3-bis(2-methoxy-4-nitro-5-sulfophenyl)-5-[(phenylamino) carbonyl]-2H-tetrazolium hydroxide) XTT test was analyzed to prove the cytotoxicity of synthesized MgO NPs toward the human melanoma cancer cell line (A375). In this study, the cells were exposed to MgO NPs at different concentrations of control, 20, 40, 60, 80, 100 and 120 μg/mL for various time intervals. The cells were made in accordance with the accrual of XTT salt, and the ratio of XTT damage via the mitochondria of the viable cells resulted in the formation of soluble formazan, which was applied to evaluate the ratio of the viable cells. The total amount of formazan that appears on the sample suspension was evaluated spectrophotometrically at an absorption frequency of 450 nm [27].

#### 2.7.3. Neutral Red Uptake (NRU)

Neutral red uptake (NRU) assay, human melanoma cancer cell line (A375) cells were exposed to various ratios of control, 20, 40, 60, 80, 100, and 120 μg/mL synthesized MgO NPs at different time intervals in 96-well plates. Continuing to the incubation time, the A375 cells were treated to a neutral red dye suspension along with 100 μL of NRU (50 μg/mL) and then the serum-free medium was diluted. Afterward, the cells were washed through PBS and the dead intake cells were also diluted in 200 μL for a constant solution. The ratio of intake neutral red through A375 effectively showed the amount of live cells [28].

#### 2.7.4. Lactase Dehydrogenase Release Assay

Lactase dehydrogenase (LDH) breakage or excretion due to the synthesized MgO NPs was evaluated with help of an LDH kit. The membrane stability of the cells after the treatment to different ratios of synthesized MgO NPs (control, 20, 40, 60, 80, 100, and 120 μg/mL) was determined by analyzing the ratio of LDH elimination. In this assay, 10 μL of the collected supernatant solution was dispersed to HBBS solution at different time periods in order to avoid impurities of phenyl red and FES suspension in culture media. The 100 mL of freshly prepared solution was moved into the separate wells and then placed in an incubation chamber under dark conditions for 30 min. Then 50 mL of 1N HCL was added to the reaction that catalyzes the lactate into pyruvate with immediate reaction to the NAD+ to NADH. The transformation of NAD+ was evaluated by absorption frequency at 340 nm. Unexposed cells were used as a control sample. For the highest elimination of LDH assessment, the cell was treated with 1% Triton X-100 for 1 h prior to the assessment and the higher amount of NAD+ reduction was calculated via UV spectrophotometer [29].

### 2.8. Genotoxic Evaluation

#### Comet Assay

The human melanoma cancer cell line (A375) was treated with different ratios of synthesized MgO NPs (Control, 20, 40, 60, 80, 100, and 120 μg/mL). The cells also treated with 3 μg/mL of H_2_O_2_ (hydrogen peroxide) were noted as a positive control sample, and untreated cells were noted as a negative control sample. Afterward, the cells were kept for incubation reaction for about 4 h and the incubated cells were washed with the help of cold PBS solution along with the trypsin-EDTA. The cells were admitted to the centrifugation process at 1500 rpm for 10 min. For the evaluation, 50 cells from each ratio were selected blindly, and the cells were viewed in 400× resolution power by using analyzing instrument (C omet 4.0 which is coupled with a fluorescence microscope connected to a CCD camera). The total amount of DNA damage and olive tail migration was mentioned as a criterion for the determination of the maximum tail DNA in every cell. The entire assay was reperformed three times to analyze the dose dependency [29].

### 2.9. Oxidative Stress Parameters

#### 2.9.1. Determination of ROS

Human melanoma cancer cell lines (A375) were added to a 96-well plate along with the different ratios of 10^4^ cells/well. After the cells were exposed to different concentrations of synthesized MgO NPs (control, 20, 40, 60, 80, 100, and 120 μg/mL) and the treated cells were kept for 6 h incubation. The MgO NPs treated cells were rinsed by using phosphate saline solution and then incubated via 20 μm (2,7-dichlorofluorescein diacetate) DCFDA dye for 1 h at 27 °C. Continuing the reaction process, the 200-μL phosphate saline solution was added, and then the fluorescence frequency was evaluated at 528 and 485 nm [30].

#### 2.9.2. Measurement of Lipid Peroxidation

Human melanoma cancer cell lines (A375) were transferred to a 96-well plate, and the fresh cells in the ratio of 10^4^ cells/well. Then the cells were allowed to grow in the plate and the cells were exposed to various ratios of obtained MgO NPs (control, 20, 40, 60, 80, 100 and 120 μg/mL) and incubated for 6 h. The treated cells were chilled with the help of PBS solution, and then the cells were rinsed with PBS solution for 10 min at 1500 rpm at 4 °C. The attained cells were sonicated at 15 W to procure the lysis cell. Then the lysate cells were filtered by lipid hydroperoxide and then mixed with the chloroform. The end suspension was exposed to ferrous ions to attain the ferric ions. The ferric ions were analyzed by using spectrophotometry at the absorption frequency of 500 nm by thiocyanatechemogen, with standard 13-HpODE solution [31].

### 2.10. Antibacterial Activity

Antibacterial activity was performed against human bacterial disease-generating pathogens such as Gram-positive bacteria, namely *S. epidermidis* MTCC 2639, *B. subtilis* MTCC 1133, and Gram-negative bacteria such as *P. aeruginosa* MTCC 2582 and *E. coli* MTCC 1692, applied on this study. The nutrient broth was procured and kept for the sterilization process. The human bacterial pathogens was inoculated separately and then the sample was incubated at 37 °C for 8 h to obtain fresh bacterial inoculums separately. The Kirby–Bauer disk diffusion assessment was used in this activity. Each bacterial sample was spread over the Muller–Hinton agar plates. The plates containing disks of 5-mm width were added on with respective NPs disk A (Control), disk B (MgO NPs in various concentrations 20, 40, 60, 80, 100, and 120 μg/mL), disk C (leaf extract), and disk D (standard antibiotics). Then, all the plates were incubated at 37 °C for 24 h, and the results were noted as a zone of inhibition [32].

## 3. Results

### 3.1. Synthesis and Physico-Chemical Characterization of MgO NPs

**Figure 1:**
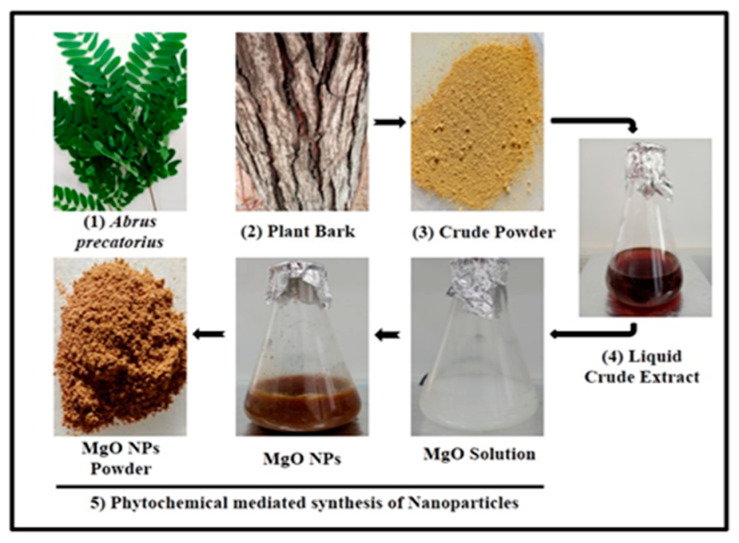
Schematic representation of the method adopted for the green synthesis of MgO nanoparticles.

**Figure 2:**
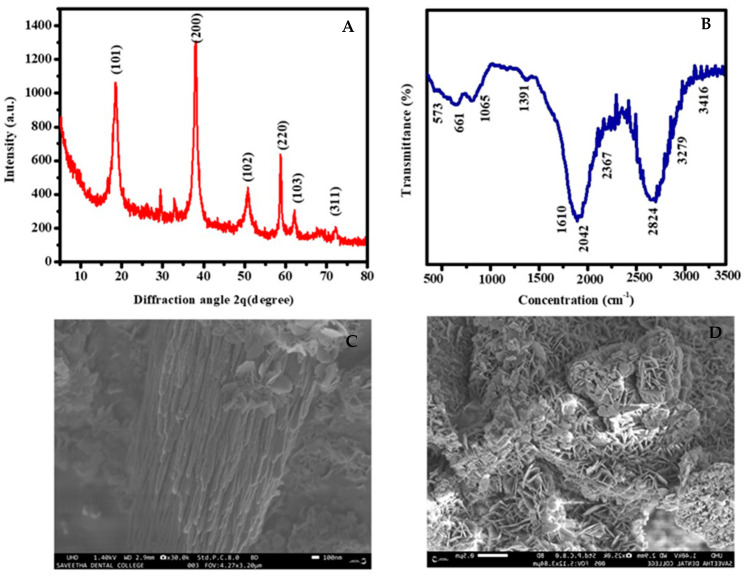
Characterization of MgO nanoparticles. (**A**) XRD, (**B**) FTIR spectrum, and (**C**,**D**) SEM images under different magnifications.

The phytochemical constituents that appear in the *A. precatorius* bark were identified as a useful substance for obtaining MgO NPs. The aqueous magnesium ions were treated with *A. precatorius* extract and were reduced, and magnesium oxide nanoparticles formed [33]. The synthesis mechanism of MgO NPs concluded through the obtained solution from brownish to dark brownish-red precipitation was revealed in Figure 1. The precipitated solution was heated to 80 °C for 3 h to attain MgO NPs. Figure 2A XRD spectrum of synthesized MgO NPs showed the presence of a sharp frequency: ~18.59° (101), ~38.04° (200), ~50.95° (220), ~62.04° (103), and ~72.44° (311).

The obtained XRD spectrum matched with the JCPDS file no. 076-0704 concluded that the particles were developed with a hexagonal, tightly packed structure (hcp), which confirmed the particles are crystalline in nature [34]. The FTIR absorption frequency, respective to the synthesized MgO NPs, was depicted in Figure 2B. The IR spectrum depicted the wavenumber at 573, 661, 1065, 1391, 1610, 2042, 2367, 2824, 3279, and 3416 cm^−1^, correspondingly. Alcohol and phenol compounds of O–H stretching were deduced from the wavelength spectrum depicted at 3279 and 3416 cm^−1^. In addition, the absorption spectrum from the wavenumber at 2824, 2367, 2042, 1610, and 1391 cm^−1^ showed the presence of C-H stretch, C=C stretch, and C=O band, due to the presence of phenol, alkynes molecules, and amide group, which has biological roles including defense responses, antitumor, and ripening fruits [35]. Moreover, a similar IR spectrum at 1065, 661, and 573 cm^−1^ reveals C-N stretching, C-Cl halogen compounds, and C-Br stretching of proteins and alkyl halides. FTIR spectrum shows the formation of dust, which might be because of the functional compounds of phytochemicals or because of the intake of moisture content. However, the closer results of moisture intake appeared in published data.

**Figure 3:**
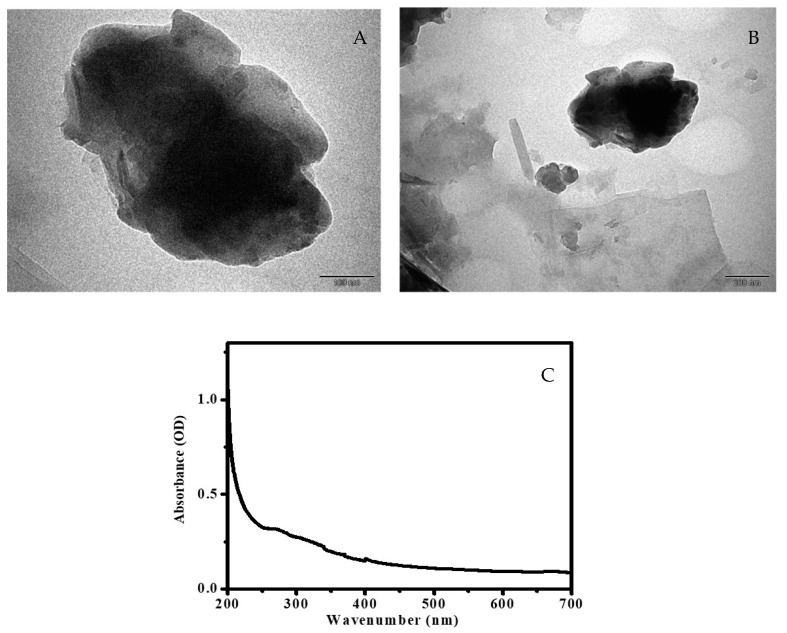
(**A**,**B**) TEM images under different resolutions and (**C**) UV-Vis spectrum.

Figure 2C,D depicts that the *A. precatorius*-associated green synthesis of MgO nanoparticles of SEM pictures concludes irregular agglomerate shape with the size scale of 100 nm. The magnesium oxide nanoparticle’s shape was determined via transmission electron microscopy (TEM), which provides clear details about the shape of the obtained nanoparticles. Figure 3A,B depicts that the obtained MgO nanoparticles have an agglomerate shape in the scale of 100–200 nm along with uniform particle distribution. The absorbance spectrum of the synthesized MgO NPs might be seen at 272 nm (Figure 3C), and the absorption wavelength of the MgO NPs was matched with the published data for confirmation [36].

### 3.2. Photocatalytic Activity

**Figure 4:**
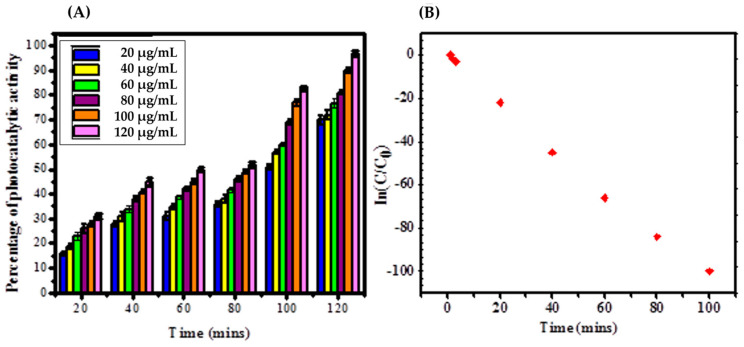
(**A**) Methylene blue dye degradation activity of MgO NPs, (**B**) Kinetic study of photo−catalytic activity of MgO NPs.

For this study, methylene blue was used as an environmental contaminating agent to evaluate the property of MgO NPs to eliminate the contaminants that appear in the wastewater for analyzing the environmental uses. Based on Figure 4A the synthesized MgO NPs revealed 30.89% photocatalytic degradation at (20 μg/mL) lower concentration whereas the higher concentration (120 μg/mL) revealed 96.78% for 120 min. From the results, the synthesized MgO NPs not only revealed a higher amount of photocatalytic degradation but also showed dose-dependent activity.

Figure 4A shows that the photocatalytic degradation of MB by synthesized MgO NPs directly revealed the sample’s spectral absorbance. The untreated MgO NPs and MB were marked as controls, and no changes were observed. Figure 4B concluded that the photocatalytic activity of synthesized MgO NPs through a plot of A°/A against time and rate constant, half-life, and R^2^ to be 9589 min^−1^, 40.29 min and 0.9989 in 40 μg/mL ratio, effectively [22,37]. The synthesized MgO NPs revealed better photocatalytic activity by direct means of the degradation of MB, which acts as a catalyst. The mechanism of photocatalytic degradation happens in the system. In the prior stage, the presence of UV light causes the MgO NPs to strongly rupture the MB via electron-hole pair development that induces the conduction band and valence band gap, which leads to coupling with the synthesized MgO NPs exterior. The electron-hole pair development captures the MB dye by developing reactive intermediates via oxidation. The presence of diluted oxygen causes the formation of O_2_ via synthesized MgO NPs electrons after its conversion to hydrogen peroxide to hydroxyl radical. Through decomposed H_2_O, the increased electron-hole pair and induced oxidative ability generate an increased hydroxyl radical [38]. The vacant oxygen species of active electron acceptors hold the light-stimulated electrons on the exterior of photocatalytic degradation, which converts O_2_ to O_2_^−^ in the exterior radical of OH− band change dye. The degradation mechanism happens in the systems. The energy of the valence band and conduction band is clearly known from the surface of MgO NPs. The entire mechanism happens on the surface of synthesized nanoparticles, and the catalysis happens in the formation of hydroxyl radicals, which results in the stimulation of methylene blue degradation, as illustrated in Figure 4A,B.

### 3.3. Antioxidant Activity

**Figure 5:**
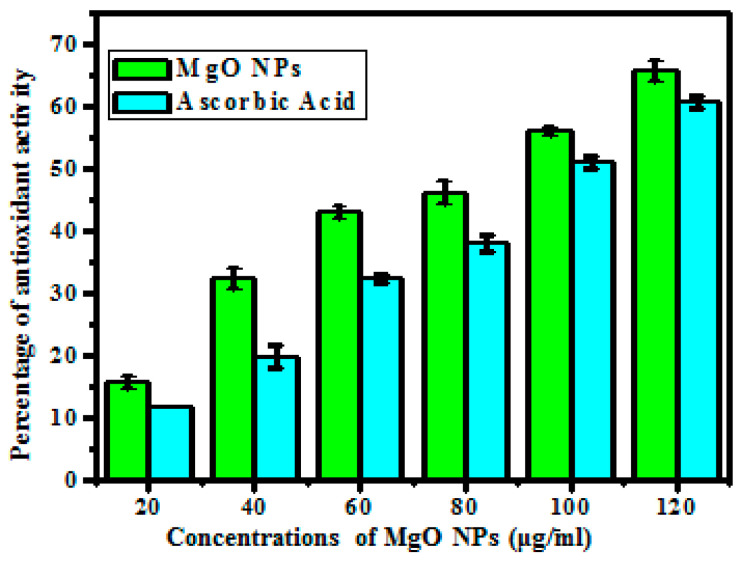
Antioxidant properties of *Abrus precatorius* bark-mediated synthesized MgO NPs and ascorbic acid.

The free radical scavenging percentage of synthesized MgO NPs was assessed via the DPPH assay, and the assay depended on the time and concentration of the reaction. The synthesized MgO NPs free radical scavenging was depicted in Figure 5 and Table 1. The obtained results showed that the low concentration (20 µg/mL) revealed 15.8% and the increased ratio (120 µg/mL) revealed 65.93% free radical scavenging, whereas the standard ascorbic acid concentration (20 µg/mL) revealed 11.66% and the increased ratio (120 µg/mL) revealed 60.86% free radical scavenging. Compared to ascorbic acid, the synthesized MgO NPs showed a better percentage of free radical scavenging. Because of the particle’s colloidal futures and exterior characteristic nature, the synthesized MgO NPs produced better results [39].

### 3.4. In Vitro Toxicity

**Figure 6:**
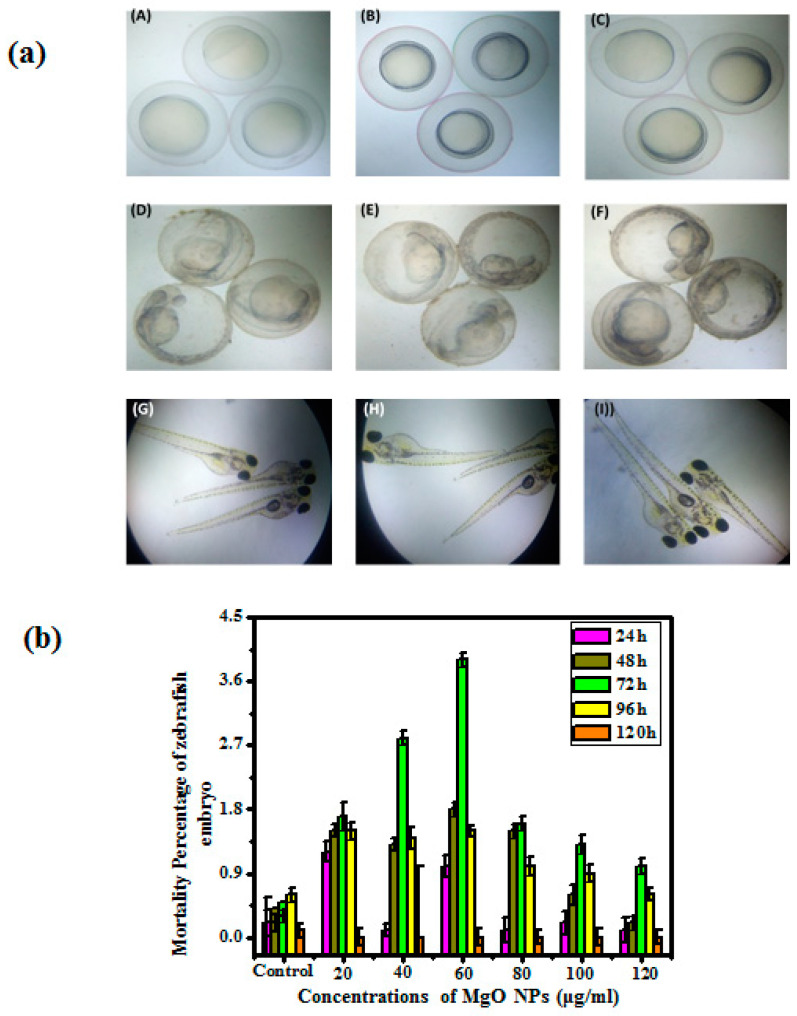
(**a**). Images representing the zebrafish embryos with hours postfertilization (hpf) (**A**) Control after 24 hpf. (**B**) MgO nanoparticles treated after 24 hpf (20 µg/mL). (**C**) MgO nanoparticles treated after 24 hpf (120 µg/mL). (**D**) Control after 48 hpf. (**E**) MgO nanoparticles treated after 48 hpf (20 µg/mL). (**F**) MgO nanoparticles treated after 48 hpf (120 µg/mL). (**G**) Control after 72 hpf. (**H**) MgO nanoparticles treated after 72 hpf (20 µg/mL). (**I**) MgO nanoparticles treated after 72 hpf (120 µg/mL). (**b**). Bar graph represents the mortality percentage of prepared MgO nanoparticles with respect to time and concentration.

The various concentrations of obtained MgO NPs dispersed in Hank’s suspension for the study of in vitro toxicity assessment. The selected zebrafish eggs were applied for a toxicity assay depending on mortality and viable eggs after the exposure of nanoparticles. Figure 6a depicted that the synthesized MgO NPs exposed eggs and matured eggs in 24 and 48 hpf seemed under 40× resolution via light microscope.

Figure 6b depicts that the synthesized nanoparticles reveal a 1.7% death rate at 20 µg/mL, whereas a 60 µg/mL concentration showed a 3.9% death rate. Then 72-h treated eggs were viewed under a microscope for visual observation of tail, head, and eye formation and deformation, whereas at 92-h and 120-h treated eggs, the majority of eggs were hatched because of nanoparticle-influenced development in zebrafish eggs. Figure 6a,b reveals that at the prior stage, the egg development and toxicological effects of synthesized MgO NPs seemed to be very low.

Figure 6b and Table 2 show that the synthesized MgO NPs showed no toxicological properties in the treated zebrafish eggs [25]. The results confirmed that a few synthesized MgO NPs exposed eggs showed delayed hatching during the earlier period of exposure. The obtained results from the zebrafish toxicological assay confirmed that no toxicity was observed in higher ratios of 100 and 200 μg/mL, which revealed a prior embryonic period and retard hatching. The retardation in the hatching period might be because of the adaptation of eggs for hatching; moreover, it is not showing any toxic effects; therefore the synthesized MgO NPs are nontoxic in nature. The in vitro toxicity study of zebrafish reveals less toxicological results concluded from reported data [36]. However, the synthesized MgO NPs is one of the essential steps for the utilization of biomedicine and are applied because of their biocompatibility.

### 3.5. Cytotoxic Evaluation

#### 3.5.1. MTT Assay

**Figure 7:**
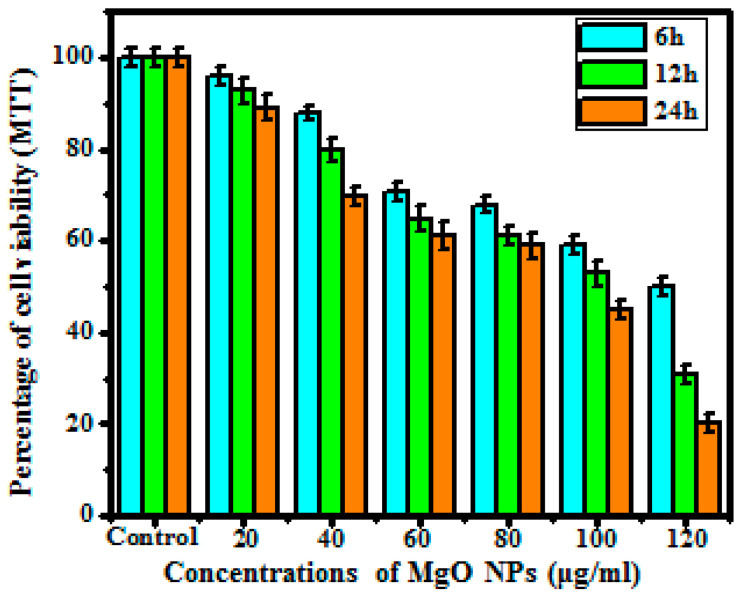
Cell viability of MgO nanoparticles as analyzed by MTT assay.

MTT assessment was implemented to determine the cell viability of A375 human melanoma cancer cells via cellular-mediated activity. Figure 7 depicts that the initial concentration at 20 μg/mL shows 96.09% of cell reduction in 6 h, whereas at 120 g/mL, it shows 89.09% of cell reduction in MTT. In increased time and concentration, the synthesized MgO NPs at 20 μg/mL reveal 49.89% cell reduction, whereas at a higher concentration of 120 μg/mL, a higher ratio of cell reduction is observed, up to 20.18% in 24 h. The obtained results show the dose-mediated activities of the obtained magnesium oxide nanoparticles toward the A375 melanoma cancer cell line. Figure 6a revealed the percentage of MTT cell reduction due to cell mortality through the treatment of synthesized MgO NPs on the A375 cell line, which is based on time-mediated activity. For confirmation, the procured results were matched with published data. Therefore, the synthesized magnesium oxide nanoparticles most effectively target the cancer cell, and the closer results are mentioned in published data [40,41].

#### 3.5.2. XTT Assay

**Figure 8:**
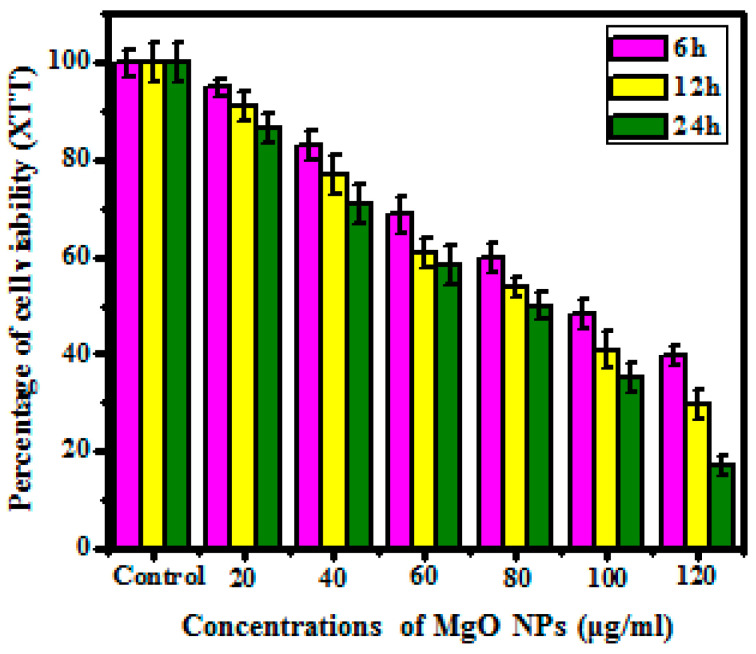
Cell viability of MgO nanoparticles as analyzed by XTT assay.

The XTT assay was a closer version of the MTT assessment where the cell viability was evaluated via XTT salt breakage into XTT formazan via mitochondrial enzymes and the alteration due to the cell viability using calorimetrically. Figure 8 reveals that the synthesized MgO NPs show ratio and time-related cytotoxic effects on the human melanoma cancer cell line (A375) [42]. From the obtained results, a 20 μg/mL concentration showed 94.89% percentage of XTT salt-reduction whereas a 120 μg/mL concentration shows a higher ratio of XTT salt reduction up to 86.67% in 6 h. Based on the result at lower concentrations, the synthesized MgO NPs does not show a higher percentage of cell reduction compared with increased concentration. The 24-h treated sample shows 39.80% salt reduction at 20 μg/mL, whereas the 120 μg/mL sample shows 17.01% cell reduction inferring that the properties of obtained magnesium oxide nanoparticles are confirmed dose- and time-mediated reaction. Hence, the obtained results concluded that the obtained magnesium oxide nanoparticles have eventually proved targeted activity against cancer cells, which does not affect normal cells in the body. Stimulated activity and cell reduction of obtained nanoparticles was confirmed via previously published data [43]. The cell mortality of the human melanoma cancer cell line (A375) might be related to the aggregation of obtained MgO NPs on the cell leading to oxidative stress-mediated apoptosis.

#### 3.5.3. NRU Assay

**Figure 9:**
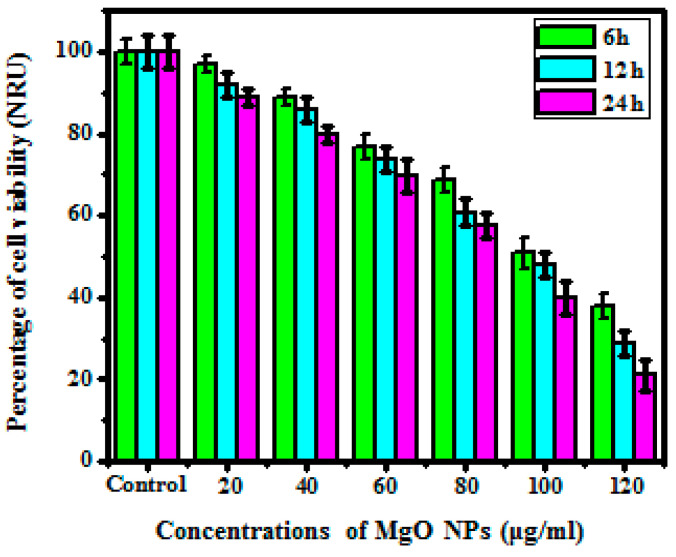
Cell viability of MgO nanoparticles as analyzed by NRU assay.

The NRU assay is utilized to determine the cytotoxic properties of the synthesized MgO NPs in human melanoma cancer cell lines by analyzing the concentration of live cells. When the live cells are exposed to the neutral red dye, the dye gets patched to the surface of the lysosome due to the various pH levels between the intracellular cytoplasm and lysosome. The viability of the cells is determined by the uptake of neutral red dye in the cell [35,44]. Figure 9 reveals that the NRU was also a concentration- and time-mediated activity. A 20 μg/mL sample exposed to A375 cells reveals 96.79% of neutral red dye absorbed, whereas a higher ratio of 120 μg/mL shows 88.89% of NRU reduction in 6 h. As a result, the 24-h treated sample at 20 μg/mL shows 37.81% cell reduction, whereas the increased concentration of 120 μg/mL shows 20.98% cell reduction, and the results confirmed the higher percentage of cell reduction. Effectively, the NRU results show that the MTT and XTT studies are much more similar to each other. This suggests that both assays, namely mitochondrial breakage and lysosomal breakage, are part of the same path that leads to cell death [45].

#### 3.5.4. LDH Release Assay

**Figure 10:**
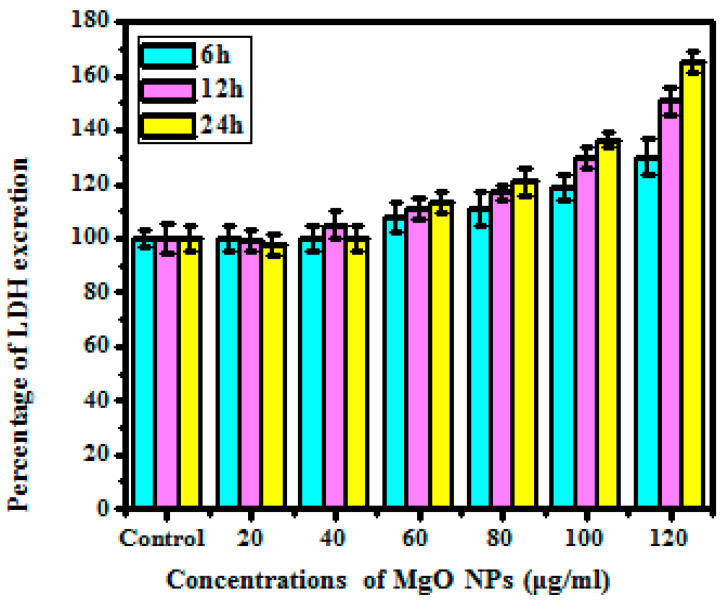
Cell viability of MgO nanoparticles as analyzed by LDH release assay.

LDH excretion assessment is a class of cytotoxic assessment in which cell membrane breakage and excretion of lactase dehydrogenase in culture media are analyzed to conclude cell mortality. Compared with previous studies, the LDH excretion assessment depicts effective results. Figure 10 shows that the lower concentration at 20 μg/mL shows no excretion of LDH in the media, whereas the higher ratio at 120 μg/mL depicts 65% of LDH excretion in the media. The total percentage of LDH excretion was directly mediated by the ratio of cell formation.

The LDH assessment related that the cell death due to the suppression of membrane solidity was effectively noted as necrosis. When the cells are exposed to the increased ratio of synthesized MgO NPs, it enhances the oxidative stress inside the cell, which leads to impediments to the membrane’s stability. The release of LDH into the culture medium speeds up the process of changing NADP to NADPH or lactate to pyruvate [46].

### 3.6. Genotoxic Assay

#### Comet Assay

**Figure 11:**
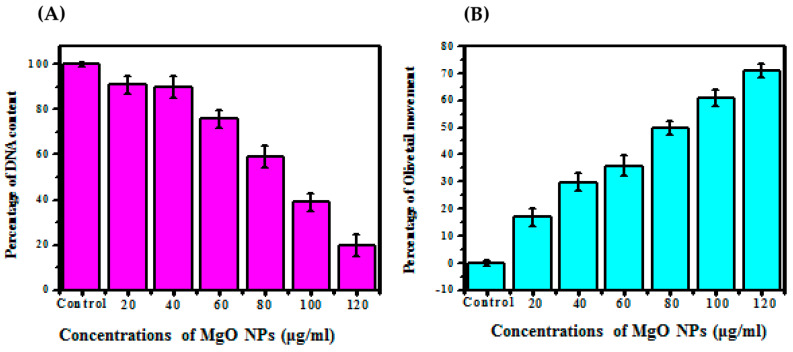
Genotoxic effect of different concentrations of MgO nanoparticles on A549 (**A**) Percentage of DNA damage, (**B**) Percentage olive tail movement.

The comet assay is an improved and ideal assay for genotoxicity caused by chemogens in cells. This test was used to determine the total tail length and also the distortion and non-formed shape depicted by the genomic components of the damaged cell when it migrates on the agarose gel. The human melanoma cancer cell line (A375) was treated with MgO NPs in this study, and the effect on DNA tail migration in agarose gel was tested. Figure 11 depicts the comet assessment results in the A375 at various concentrations (control, 20, 40, 60, 80, 100, and 120 μg/mL) [47]. According to the results, a lower concentration of 20 μg/mL results in 90.98% breakage of genomic DNA, whereas an increased concentration results in 19.98% breakage of genomic DNA. Based on genomic DNA damage, Figure 11B concluded that, at a lower ratio, 20 μg/mL depicts 16.9% of olive tail migration, whereas 120 g/mL depicts 70.98% of olive tail migration [48]. For eventual proof, the obtained results were compared with previously published data, revealing 22% and 5.37% of the olive tail movement in 20 μg/mL and 120 μg/mL, depending on the ratio-mediated activity, and the obtained results showed the enhanced ratio of DNA damage and olive tail movement.

The increased ratio of MgO NPs in the human melanoma cancer cell line (A375) causes increased DNA damage, which leads to cell death. The DNA strand breakage with the exposure of synthesized MgO NPs depicts that the cell has gone into apoptosis. The MgO NPs effectively stimulate natural ROS at the exterior position, which effectively initiates the formation of free radicals when it’s attached to lipids and protein substances, leading to cellular breakage or necrosis [49].

### 3.7. Measurement of Oxidative Stress Parameters

The genotoxic assay of the synthesized MgO NPs exposed to A375 cell lines depicts an effective concentration of genomic damage, which infers that cell death because of genetic damage depends on the apoptotic pathway caused by intracellular ROS. The oxidative stress components of synthesized MgO NPs treated in the A375 human melanoma cancer cell line were examined by using this assay. The major component targeted in this study was used to measure the oxidative stress components, i.e., the intracellular ROS, which effectively confirmed that the cell mortality resulted in ROS−related apoptosis. The lipid peroxidation shows the cell has gone through necrosis and the absence of membrane stability [50].

#### 3.7.1. Measurement of Intracellular ROS

The intracellular ROS measurement gives acceptable facts about oxidative stress-related apoptosis inside the cell. Based on the higher absorption of cell-penetrable DCFDA dye, Table 3 depicts the better ratio of ROS formation inside the cell. Table 3 shows that the treatment of A375 cell lines with synthesized MgO NPs increased intracellular ROS formation by 51% in 24 h. The genesis of random ROS is because of the chemical and exterior features of synthesized MgO NPs on the A375 human melanoma cancer cell line. This ROS surrounding the exterior of synthesized MgO NPs might further induce the formation of free radicals due to the activation of intracellular constituents, which leads to damage to mitochondria. The intracellular ROS formation due to the initiation of the oxidase enzyme (NADPH) induces the formation of the superoxide anion O^2^ in the membrane of phagocytic cells. This intracellular chemical species, like DNA, oxidizes and reduces to lesser macromolecules, resulting in oxidative stress-mediated apoptosis [51].

#### 3.7.2. Measurement of Lipid Peroxidation

Lipid peroxidation is applied to understand oxidative stress-related necrosis due to the impediment of membrane stability. Lipid peroxidation is calculated via the concentration of hydrogen peroxide-induced elimination. Table 3 reveals that the synthesized MgO NP-exposed A375 human melanoma cancer cell lines depicted no notable ratio of lipid peroxidation at 20 μg/mL, whereas an increased ratio of 120 μg/mL reveals 6.1 ± 0.9 lipid peroxidation in 24 h [52,53,54]. The increased concentration of synthesized MgO NPs alters the electron-hole pair that enhances ROS genes, which leads to the formation of free radicals connect with biomolecule breakage of proteins and lipids to develop instability of membrane stability [55,56,57,58,59,60].

### 3.8. Antimicrobial Activity

**Figure 12:**
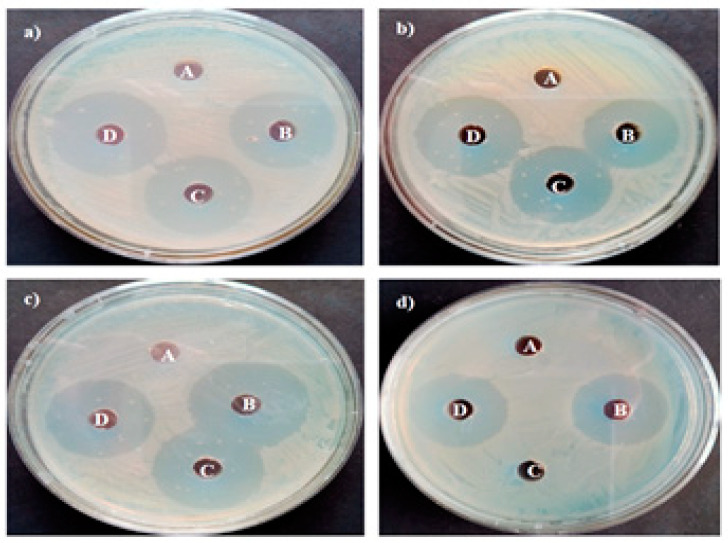
Antimicrobial activity of synthesis of MgO NPs (A: DMSO, B: MgO NPs, C: *Abrus precatorius* bark extract and D: standard antibiotics), and (**a**) *S. epidermidis* MTCC 2639, (**b**) *B. subtilis* MTCC 1133, (**c**) *P. aeruginosa* MTCC 2582, and (**d**) *E. coli* MTCC 1692.

The obtained results are depicted in Figure 12 and Table 4. The *A. precatorius*-mediated synthesized MgO NPs demonstrated the antimicrobial properties of synthesized nanoparticles by observing the inhibition towards Gram-positive and Gram-negative bacteria. In this study, the zone of inhibition formation was noted towards Gram-positive bacteria like *S. epidermidis* MTCC 2639 (20 ± 0.26 mm) and *B. subtilis* MTCC 1133 (31 ± 0.25 mm) and against Gram-negative bacteria like *P. aeruginosa* MTCC 2582 (22 ± 0.50 mm) and *E. coli* MTCC 1692 (30 ± 0.10 mm) (Figure 11). Differentiating from all the samples, the maximum ratio of zone inhibition is noted at *B. subtilis* MTCC 1133 (31 ± 0.25 mm) and *E. coli* MTCC 1692 (30 ± 0.10 mm). Hence, this assay concludes with a better zone of inhibition [61,62].

Previous research found that metal oxide nanoparticles broke the cell membrane and ruptured the cell inside. It was concluded that the removal of H_2_O_2_ is an alternative substitution for antimicrobial activity [63,64,65]. This hypothesis also needs experimental proof because of the low ratio of synthesized MgO NPs, which do not efficiently develop H_2_O_2_. A low concentration of synthesized MgO NPs does not generate a toxic effect in the human system. The day-to-day intake of magnesium via food was essential for metabolic pathways. The synthetic MgO was known for its ability to form a barrier against *E. coli* intestinal tract breakage. When magnesium reacts with acid and generates Mg^2+^ ions, the pH in the stomach rises from 2 to 5. For these activities, a few enzymes are essential, such as carboxyl peptidase, carbonic anhydrase, and alcohol dehydrogenase, which are essential for alcohol digestion and carbohydrate digestion, respectively. The cell mortality pathway of synthesized MgO NPs is (1) the genesis of ROS and (2) stimulation of cell mortality, resulting in the rupture of cellular compounds like proteins, lipids, and DNA. Furthermore, the synthesized MgO NPs induce toxic properties against bacterial cells, which result in cellular mortality [66].

## 4. Conclusions

The purpose of this study was to examine the long-term applications of obtained magnesium oxide nanoparticles (NPs) as an effective anticancer drug. MgO NPs are synthesized by using *A. precatorius* bark extract. X-ray diffraction analysis determined that MgO NPs have a polycrystalline wurtzite structure. The functional components of the synthesized nanoparticles are determined through FTIR analysis. The SEM and TEM results concluded that the obtained nanoparticles are spherical in shape. The synthesized MgO NPs reveal higher photocatalytic activity and antioxidant activity. The MgO NPs safety nature was confirmed by zebrafish viability, and the result concluded the particles are effectively used as a potential drug for the human system. The cytotoxicity of synthesized MgO NPs was investigated in order to determine the cell-mortality cycle by using the A375 human melanoma cancer cell line. The MTT, XTT, and NRU assays depend on the mitochondrial and lysosomal cytotoxic assay, which confirmed that the cytotoxicity activity of synthesized MgO NPs is a time- and dose-mediated activity. The LDH assay was used to determine the integrity of the cell membrane. The NRU assay showed an increase in lactase dehydrogenase excretion at a higher ratio. The comet assay depicts the synthesized MgO NPs-mediated ROS formation that can induce DNA damage, which results in apoptosis in the human melanoma cancer cell line (A375). In conclusion, this study confirmed that the green-mediated obtained MgO NPs could be a potential anticancer drug, and this research not only aimed to obtain MgO NPs via phytochemical components of *A. precatorius* bark extract but also to perceive a substitutive method for the genesis of an efficient anticancer drug for potential biomedical applications.

## Figures and Tables

**Table 1:** Antioxidant activity of synthesized MgO NPs synthesized from *Abrus precatorius* bark extract.

Concentration (µg/mL)	Antioxidant (%)	
	Percentage of DPPH Inhibition (Absorbance 517 nm)	
	Ascorbic Acid	MgO NPs
20	11.66	15.8
40	19.93	32.33
60	32.33	43.16
80	38.16	46.2
100	51.00	56.06
120	60.86	65.93

**Table 2:** In vitro toxicity assay of zebrafish death percentage, *A. precatorius* bark extract synthesized MgO NPs against various time and dosage manner.

**MgO NPs**	**Concentration** **(µg/mL)**	**Mortality (%)**				
		**24 h**	**48 h**	**72 h**	**96 h**	**120 h**
	Control	0.2 ± 0.2	0.2 ± 0.4	0.3 ± 0.5	0.6 ± 0.6	0.1 ± 0.4
	20	1.2 ± 0.4	1.5 ± 0.3	1.7 ± 0.4	1.5 ± 0.5	0.0 ± 0.5
	40	0.1 ± 0.6	1.3 ± 0.2	2.8 ± 0.4	1.4 ± 0.9	0.0 ± 0.4
	60	1.0 ± 0.3	1.8 ± 0.4	3.9 ± 0.1	1.5 ± 0.3	0.0 ± 0.2
	80	0.1 ± 0.3	1.5 ± 0.8	1.6 ± 0.3	1.0 ± 0.2	0.0 ± 0.4
	100	0.2 ± 0.5	0.6 ± 0.7	1.3 ± 0.2	0.9 ± 0.1	0.0 ± 0.1
	120	0.1 ± 0.7	0.2 ± 0.3	1.0 ± 0.8	0.6 ± 0.5	0.0 ± 0.3

**Table 3:** *Abrus precatorius* bark extract synthesized MgO NPs effect on the ROS generation and lipid peroxidation in A549 cells after 24 h.

Concentration	ROS Generation (%)	Hydrogen Peroxide Concentration (n/mol)
Control	100 ± 00	1.0 ± 0.9
20 µg/mL	115 ± 0.1	1.5 ± 0.3
40 µg/mL	129 ± 0.7	2.3 ± 0.6
60 µg/mL	134 ± 0.5	3.4 ± 0.7
80 µg/mL	143 ± 0.7	4.5 ± 0.2
100 µg/mL	156 ± 0.5	5.6 ± 0.4
120 µg/mL	161 ± 1.8	6.1 ± 0.9

**Table 4:** Antibacterial activity of MgO NPs studied towards various pathogens.

Microorganisms	Zone of Inhibition (Mean ± SD (mm))			
	DMSO	Leaf Extract	MgO NPs	Standard Antibiotics
*S. epidermidis* MTCC 2639	--	10 ± 0.12	20 ± 0.26	20 ± 0.06
*B. subtilis* MTCC 1133	--	16 ± 0.30	31 ± 0.25	28 ± 0.99
*P. aeruginosa* MTCC 2582	--	12 ± 0.49	22 ± 0.50	21 ± 0.90
*E. coli* MTCC 1692	--	15 ± 0.01	30 ± 0.10	29 ± 0.89

## Data Availability

Not applicable.
